# Pulmonary Vasculature Responsiveness to Phosphodiesterase-5A Inhibition in Heart Failure With Reduced Ejection Fraction: Possible Role of Plasma Potassium

**DOI:** 10.3389/fcvm.2022.883911

**Published:** 2022-05-26

**Authors:** Luca Monzo, Adrian Reichenbach, Hikmet Al-Hiti, Ivana Jurcova, Zuzana Huskova, Josef Kautzner, Vojtech Melenovsky

**Affiliations:** ^1^Institute for Clinical and Experimental Medicine (IKEM), Prague, Czechia; ^2^Department of Clinical Internal, Anesthesiological and Cardiovascular Sciences, Sapienza University, Rome, Italy

**Keywords:** pulmonary hypertension, heart failure, phospodiesterase inhibitors, potassium, pulmonary vascular resistance

## Abstract

**Introduction:**

Phosphodiesterase-5a inhibition (PDE5i) leads to favorable changes in pulmonary hemodynamic and cardiac output (CO) in patients with advanced heart failure (HF) and reduced ejection fraction (HFrEF). The hemodynamic response to PDE5i could be heterogeneous and the clinical variables associated with these changes are scarcely investigated.

**Materials and Methods:**

Of 260 patients with advanced HFrEF referred for advanced therapies [cardiac transplant/left ventricular assist device (LVAD)], 55 had pulmonary hypertension (PH) and fulfilled the criteria for the PDE5i vasoreactivity test. Right heart catheterization (RHC) was performed as a part of clinical evaluation before and after 20-mg intravenous sildenafil. Absolute and relative changes in pulmonary vascular resistance (PVR) were evaluated to assess hemodynamic response to PDE5i. Clinical, biochemical, and hemodynamic factors associated with PVR changes were identified.

**Results:**

Sildenafil administration reduced PVR (− 45.3%) and transpulmonary gradient (TPG; − 34.8%) and increased CO (+ 13.6%). Relative change analysis showed a negative moderate association between baseline plasma potassium and changes in PVR (*r* = − 0.48; *p* = 0.001) and TPG (*r* = − 0.43; *p* = 0.005) after PDE5i. Aldosterone concentration shows a direct moderate association with PVR changes after PDE5i. A significant moderate association was also demonstrated between CO improvement and the severity of mitral (*r* = 0.42; *p* = 0.002) and tricuspid (*r* = 0.39; *p* = 0.004) regurgitation.

**Conclusion:**

We identified plasma potassium, plasma aldosterone level, and atrioventricular valve regurgitations as potential cofounders of hemodynamic response to acute administration of PDE5i. Whether modulation of potassium levels could enhance pulmonary vasoreactivity in advanced HFrEF deserves further research.

## Introduction

Pulmonary hypertension (PH) is highly prevalent in patients with heart failure (HF) (1) and impacts detrimentally right ventricular (RV) function, exercise capacity, and survival (2, 3). PH in HF occurs due to the transmission of high left ventricular (LV) filling pressure into pulmonary vessels and due to an increase of pulmonary vascular resistance (PVR) (4). The resultant combined pre- and post-capillary PH can be particularly detrimental in patients with HF considered for left-sided mechanical support or heart transplant (5). Despite left ventricular assist device (LVAD), implantation significantly improves survival in end-stage HF (6), it is frequently complicated by right heart failure (RHF) (7) leading to a higher risk of adverse outcomes (8). Increased RV afterload due to high PVR is a critical determinant of acute graft failure immediately after transplantation (9), and it also contributes to RV dysfunction after LVAD (9, 10).

Phosphodiesterase-5a inhibitors (PDE5is) are selective pulmonary vasodilators that attenuate the degradation of cyclic guanosine monophosphate (cGMP) and are clinically used for the treatment of pulmonary arterial hypertension (11, 12). In some centers, PDE5is are used off-label to mitigate the detrimental impact of PH in advanced HF with reduced ejection fraction (HFrEF) patient prior heart transplantation or LVAD implantation (13–15). However, there are no randomized clinical trials to support this approach, which is still a subject of debate (4, 16, 17). In some transplant centers, such as ours, PDE5i may be used acutely for the testing of reversibility of increased PVR (18, 19).

In our practice, we noticed that acute hemodynamic response to PDE5i infusion could be heterogeneous, with some patients responding remarkably well in terms of PVR reduction (due to profound changes in its components, cardiac output (CO), and transpulmonary gradient [TPG]) (20), while others may have a small or even no response (21). To date, little research has been done about clinical variables that might influence hemodynamic response to PDE5i in patients with HF. If such variables are identified, then PDE5i therapy could be tailored to patients who benefit most, without exposing non-responders to potential side effects, such as hypotension or bleeding (22). Alternatively, factors associated with a poor response may be addressed to improve PDE5i responsiveness.

Given these premises, we explored clinical factors associated with acute hemodynamic response to PDE5i in an advanced HFrEF cohort.

## Materials and Methods

### Study Population

The study retrospectively enrolled patients with chronic (> 6 months) advanced HFrEF (EF < 40%) electively hospitalized at the Institute for Clinical and Experimental Medicine (IKEM) in Prague between April 2017 and October 2019 for consideration of heart transplant or LVAD implantation. Patients with acute ischemia, uncontrolled cardiac arrhythmia, reversible cardiac dysfunction, active malignancy, endocrine disease, pre-existing PDE5i therapy, nitrate use, chronic or acute infection, or those unwilling to participate were excluded. Patients with hypervolemia on admission were enrolled in the study only if reached a normovolemic state (mean central vein pressure < 10 mmHg) after intravenous diuretics treatment. The testing of hemodynamic response to intravenous sildenafil was performed clinically as it is the current standard for our clinical evaluation protocol for pre-transplant assessment; as such no placebo control was administered. According to criteria used at our institution, indications for the PDE5i vasoreactivity test were increased PVR (> 4 Wood units) or TPG (> 15 mmHg) and systolic blood pressure > 90 mmHg. This study conforms to the declaration of Helsinki and was approved by the local ethics committee that did not considered it as a clinical trial. All patients signed informed consent with the procedure and with research data collection.

### Study Protocol and Measures

Patients underwent, on the same day, morning fasting blood sampling for laboratory analysis, clinical history review, physical examination, echocardiography, ECG, and right heart catheterization (RHC). Echocardiographic exams were performed by an experienced physician, in the left lateral decubitus position with a commercially available standard ultrasound scanner (Vivid 7, General Electric Medical Systems, Wauwatosa, Wisconsin) using a 2.5 MHz phased-array transducer. LV function and dimensions were measured according to contemporary recommendations (23). Mitral and tricuspid regurgitation were assessed semiquantitatively and expressed in 4 grades according to current guidelines (24). RV function was evaluated by tricuspid annular plane systolic excursion (TAPSE) and systolic RV tissue velocity (S’-TDI). RV dysfunction was defined as a TAPSE < 17 mm or an S’-TDI < 9.5 cm/s (25). Body weight was measured by using an electronic scale (HBF-510W, Omron, Japan).

Right heart catheterization was performed after non-invasive examinations in the supine position using a 7F balloon-tipped triple-lumen Swan-Ganz catheter (Braun Melsungen AG, Germany) *via* right internal jugular vein and fluoroscopic guidance. Transducers were balanced by determining zero level at the mid-axillary line. Pressure waveforms were recorded as the average of at least 3 measurements and annotated by an invasive hemodynamic module (Mac-Lab, GE Healthcare, United States) and were measured in right atrium (RA), RV, and pulmonary artery (PA). The wedge position of the Swan-Ganz catheter was confirmed by X-ray. The value of PA wedge pressure (PAWP) was assessed at end-expiration with the balloon-tipped catheter at a steady state with the patient in a supine position (26). Cardiac output was measured by thermodilution as the average of at least 3 measurements with < 10% variance. TPG was computed by subtracting the PAWP from the mean PA pressure. PVR was calculated by dividing the TPG by the CO. PH was defined as a PA mean pressure > 20 mmHg (27). The effect of sildenafil on CO, PVR, and TPG was computed as absolute differences (post-PDE5i value − baseline) and as percent change from baseline (relative change: [(post-PDE5i value − baseline)/baseline value] × 100. Systemic blood pressure was measured after 10 min of rest in the supine position using an automated oscillometric monitor. RHC with hemodynamic recording was repeated 10 min after the administration of 20 mg of sildenafil citrate (Pfizer, New York, NY, United States) into a central vein.

### Data Analysis

Data are shown as mean ± standard deviation (SD) or median and [25th–75th interquartile range (IQR)] for continuous variables (according to distribution) and total count (n) with proportion (%) for categorical variables. Normality was assessed using the Shapiro-Wilks test. Significance of changes within subjects was tested using paired *t*-test or McNemar’s test for paired comparisons as appropriate. For abnormally distributed data, Wilcoxon signed-rank test was used. The association between baseline prognostic clinically relevant covariates and hemodynamic changes induced by PDE5i administration was assessed by linear or logistic regression as appropriate. A two-tailed value of *p* < 0.05 was considered statistically significant. All analyses were performed using JMP pro 15.0 statistical software (SAS Institute, Inc., Cary, NC, United States).

## Results

A total of 260 consecutive patients referred for advanced HF therapies (cardiac transplant/LVAD) evaluation in our center were considered for the study. Of these, 55 patients fulfilled the criteria for hemodynamic testing of PVR reversibility (see section “Materials and Methods”). Baseline clinical characteristics of the population are summarized in Table 1. Our population predominantly consisted of severely symptomatic [71% New York Heart Association (NYHA) III class; 13% NYHA IV class] middle-aged male patients, on guideline-directed medical therapy at maximally tolerated doses and equally divided between ischemic and non-ischemic HF. In patients with non-ischemic HF, the etiology was mainly idiopathic dilated cardiomyopathy (80% of cases). Two-thirds of patients (65%) displayed RV dysfunction, and all of them had PH.

**TABLE 1:** Baseline characteristics.

Clinical characteristics	
Age, years	57.2 ± 10.8
Male, *n* (%)	47 (85)
Body mass index, kg/m^2^	27.3 ± 3.7
NYHA class, *n* (%): II; III; IV	9 (16); 39 (71); 7 (13)
Heart failure etiology, *n* (%): Ischemic; non-ischemic	28 (51); 27 (49)
Diabetes mellitus, *n* (%)	23 (43)
Atrial fibrillation, *n* (%)	5 (9)
Laboratory examinations	
Hemoglobin, g/L	141.7 ± 20.6
Sodium, mmol/L	137.8 ± 3.6
Potassium, mmol/L	4.3 ± 0.4
Creatinine, μmol/L	132.4 ± 40.7
BNP, ng/L	1,148 [507; 1,781]
Renin, ng/L	113 [54; 309]
Aldosterone, pmol/L	792 ± 567
Echocardiography	
LV end-diastolic diameter, mm	72.5 ± 9.1
LV end-systolic diameter, mm	64.7 ± 10.6
LV ejection fraction,%	21.5 ± 4.3
Left atrial volume index, mL/m^2^	64.9 ± 20.1
Mitral regurgitation grade, *n* (%): 0–2; 3–4	22 (39); 33 (61)
Tricuspid regurgitation grade, *n* (%): 0–2; 3–4	39 (71); 16 (29)
Right ventricular dimensions, mm: RVD1; RVD2; RVD3	47.5 ± 6.5; 34.0 ± 4.9; 87.9 ± 6.4
TAPSE, mm	15.6 ± 3.8
Tricuspid annulus S’-TDI, cm/s	7.6 ± 2.3
Therapy	
Furosemide, *n* (%)	53 (96)
Furosemide daily dose, mg/24 h	126 ± 104
ACEi, ARB or ARNI, *n* (%)	32 (58)
MRA, *n* (%)	48 (87)
Betablocker, *n* (%)	48 (87)

*ACEi, angiotensin converting enzyme inhibitor; ARB, angiotensin receptor blocker; ARNI, angiotensin receptor neprilysin inhibitor; BNP, brain natriuretic peptide; MRA, mineralocorticoid receptor antagonist; LV, left ventricular; NYHA, New York Heart Association; RVD, right ventricular dimension; TAPSE, tricuspid annular plane systolic excursion; S’-TDI, peak systolic velocity of the tricuspid annulus by tissue Doppler imaging.*

### Hemodynamic Effects of PDE5i

**FIGURE 1:**
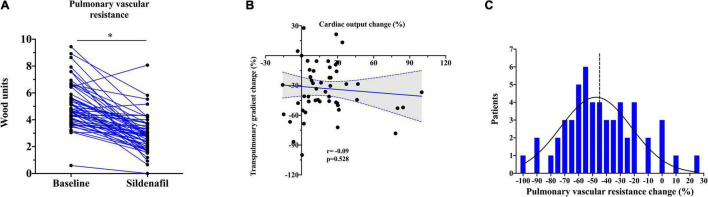
**(A)** Changes in pulmonary vascular resistance (PVR) after acute PDE5i administration. The asterisk stands for *p* < 0.001. **(B)** Correlation between relative transpulmonary and cardiac output change after PDE5i infusion. **(C)** Distribution of sildenafil response in terms of PVR in our sample. The dashed line represents the median.

At baseline, patients showed increased PA mean pressure, RA mean pressure, PVR, and PAWP. Intravenous administration of 20 mg of sildenafil was led to significant reduction in RA pressure (− 43.2 ± 9.8%) and PAWP (− 15.9 [2.5; 38.4]%). RA/PAWP pressure ratio, a global RV hemodynamic performance indicator, was significantly decreased (− 31.1 ± 30.2%), with a concurrent decrease in RV afterload (PA mean pressure − 25.4 ± 19.8%; all *p* < 0.01; Table 2). Sildenafil also led to a profound change in PVR (− 42.0 ± 25.7%, *p* < 0.001; Figure 1A) and in its components (TPG − 33.0 ± 26.5%; CO + 13.6 [1.9; 29.1]%, both *p* < 0.01). CO change was mainly driven by an increase in stroke volume (+ 6.5 [2.4; 14.2] ml; *p* < 0.01) rather than a modification in heart rate (0 [0; − 5] bpm; *p* = 0.07).

**TABLE 2:** Effect of sildenafil on hemodynamic.

	Baseline	After sildenafil	*p*-value
**Right heart catheterization**			
Cardiac output—L/min	3.70.8	4.30.8	<0.001
PVR—WU	5.11.6	2.91.4	<0.001
Transpulmonary pressure gradient—mmHg	17.94.1	11.94.5	<0.001
RA mean pressure—mmHg	8.23.4	5.13.4	<0.001
RV maximum pressure—mmHg	62.913.6	45.416.3	<0.001
RV minimum pressure—mmHg	3.53.1	1.53.0	<0.001
RV end-diastolic pressure—mmHg	11.64.2	8.66.2	0.002
PA systolic pressure—mmHg	64.213.2	47.315.5	<0.001
PA diastolic pressure—mmHg	29.57.5	20.96.7	<0.001
PA mean pressure—mmHg	42.48.6	31.49.9	<0.001
PA wedge pressure mean—mmHg	24.57.1	19.47.8	<0.001
Mean blood pressure—mmHg	88.611.9	81.513.9	<0.001
Heart rate—bpm	82.314.0	80.414.1	0.080
SVR—WU	22.85.6	18.34.9	<0.001
PVR/SVR ratio	0.220.06	0.160.07	<0.001

*PA, pulmonary artery; PVR, pulmonary vascular resistance; RA, right atrial; RV, right ventricular; SVR, systemic vascular resistance; WU, Wood units.*

Interestingly, PDE5i-induced changes in TPG and CO were unrelated, i.e., some patients showed a large increase in CO with minimal change in TPG, and vice versa, indicating heterogeneity of response (Figure 1B). In the systemic circulation, PDE5i significantly reduced the mean blood pressure (− 7.4 [−2.2; − 15.1]%) and the systemic vascular resistance (SVR; − 19.1 [ − 5.1; − 30.7]%). Vasodilation in the systemic circulation was less pronounced than in the pulmonary vascular bed as represented by a significant reduction in PVR/SVR ratio (− 31.9 [ − 2.1; − 47.9]%, *p* < 0.01).

### Clinical Variables and PDE5i Response

After PDE5i administration, PVR was reduced in average of − 45.3% (− 2.0 WU) when compared to baseline (Figure 1C). Concurrently, CO was increased on average of + 13.6% (+ 0.5 L/min) and TPG was reduced to − 34.8% (− 6.0 mmHg). PAWP showed generally a consistent reduction after PDE5i infusion, except in 10 patients (19%) who showed on the contrary a rise in PAWP. The median increase was usually mild (3 [2; 5] mmHg).

**FIGURE 2:**
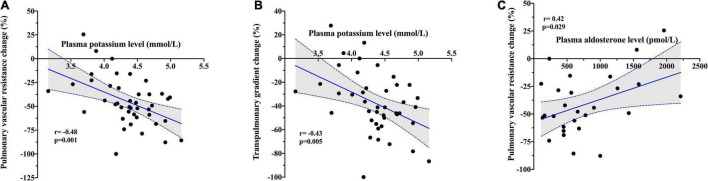
Correlation between baseline plasma potassium level and **(A)** relative change in pulmonary vascular resistance and **(B)** relative change in transpulmonary gradient after PDE5i. **(C)** Correlation between baseline plasma aldosterone level and relative change in pulmonary vascular resistance after PDE5i.

**FIGURE 3:**
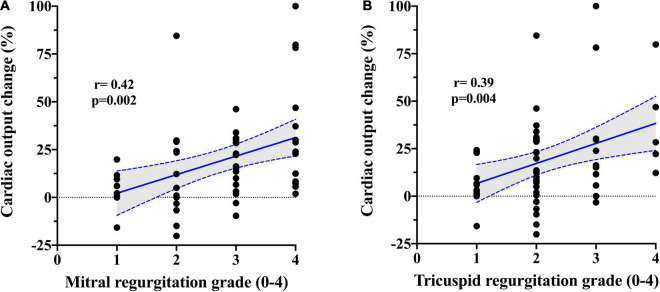
Correlation between PDE5i-induced change in cardiac output and **(A)** mitral and **(B)** tricuspid regurgitation severity.

Baseline plasma potassium level showed a negative moderate association in both absolute (*r* = − 0.45; *p* = 0.002) and relative (*r* = − 0.48; *p* = 0.001) changes in PVR after PDE5i (Table 3 and Figure 2A) and TPG change (*r* = − 0.43; *p* = 0.005; Figure 2B). Similarly, plasma potassium was inversely associated with relative changes in SVR (*r* = − 0.36; *p* = 0.018). A mild association was found between furosemide daily dose and pulmonary vasoreactivity (*r* = 0.35; *p* = 0.009). Aldosterone concentration showed a direct moderate association with PVR relative change after PDE5i (*r* = 0.42; *p* = 0.029; Figure 2C) but was inversely associated with baseline plasma potassium level (*r* = − 0.43; *p* = 0.025; Supplementary Figure 1). A significant moderate association was also demonstrated between CO improvement and the severity of mitral (*r* = 0.42; *p* = 0.002) and tricuspid (*r* = 0.39; *p* = 0.004) regurgitation (Figure 3).

**TABLE 3:** Correlation between baseline clinical variables and absolute and relative PVR change after sildenafil.

Parameters	PVR change (absolute)		PVR change% (relative)	
		
	Correlation coefficient [95% CI]	*p*-value	Correlation coefficient [95% CI]	*p*-value
Age, years	0.11 [−0.15; 0.37]	0.402	0.08 [−0.19; 0.34]	0.567
Body mass index, kg/m^2^	0.26 [−0.01; 0.49]	0.056	0.08 [−0.18; 0.34]	0.549
NYHA class, I–IV	−0.13 [−0.39; 0.16]	0.372	0.02 [−0.26; 0.30]	0.884
HF duration, years	0.27 [0.01; 0.51]	0.046	0.19 [−0.08; 0.44]	0.157
Sodium, mmol/L	−0.09 [−0.38; 0.22]	0.567	−0.26 [−0.52; 0.04]	0.095
Potassium, mmol/L	−0.45 [−0.66; −0.17]	0.002	−0.48 [−0.68; −0.21]	0.001
BNP, ng/L	−0.27 [−0.50; −0.04]	0.047	−0.07 [−0.33; 0.19]	0.601
Aldosterone, pmol/L	0.36 [−0.01; 0.65]	0.063	0.42 [0.04; 0.69]	0.029
Renin, ng/L	0.33 [−0.12; 0.66]	0.145	0.31 [−0.14; 0.65]	0.171
PAWP, mmHg	−0.06 [−0.32; 0.20]	0.644	0.03 [−0.24; 0.29]	0.837
PA pressure (mean), mmHg	−0.29 [−0.52; −0.02]	0.031	−0.02 [−0.29; 0.24]	0.858
RV-EDP, mmHg	−0.07 [−0.33; 0.19]	0.598	0.03 [−0.23; 0.29]	0.811
RV maximum pressure, mmHg	−0.09 [−0.35; 0.18]	0.506	0.10 [−0.17; 0.36]	0.457
LVEF,%	−0.07 [−0.33; 0.19]	0.598	−0.11 [−0.36; 0.16]	0.420
TAPSE, mm	0.12 [−0.16; 0.39]	0.396	−0.13 [−0.39; 0.15]	0.364
Mitral regurgitation, grade 1–4	−0.25 [−0.48; 0.02]	0.066	−0.15 [−0.40; 0.12]	0.279
Tricuspid regurgitation, grade 1–4	−0.17 [−0.41; 0.10]	0.218	−0.18 [−0.43; 0.08]	0.172
Furosemide daily dose, mg/24h	0.26 [−0.01; 0.49]	0.051	0.35 [0.09; 0.56]	0.009

*BNP, brain natriuretic peptide; HF, heart failure; LVEF, left ventricular ejection fraction; NYHA, New York Heart Association; PA, pulmonary artery; PAWP, pulmonary artery wedge pressure; PVR, pulmonary vascular resistance; RV, right ventricle; RV-EDP, right ventricular end-diastolic pressure; TAPSE, tricuspid annular plane systolic excursion.*

Patients with increased PAWP after sildenafil showed a higher relative drop in PVR when compared to patients with a reduction in PAWP (− 54.0 ± 17.4% vs. − 39.3 ± 26.6%; *p* = 0.04). Baseline predictors of this kind of response had higher BNP, lower aldosterone, and a more severe degree of tricuspid regurgitation (Supplementary Table 1).

Left and right systolic functions, HF severity and duration, the use and the dose of beta-blockers, renal function, pulmonary, and RV hemodynamic were mostly unrelated to relative changes in pulmonary vasoreactivity.

## Discussion

In our study, we investigated clinical variables possibly associated with a better hemodynamic response to PDE5i in terms of PVR change and its components (TPG and CO). Specifically, we showed that a concentration of plasma potassium at the lower edge of normality has a moderate strength in predicting impaired pulmonary vasodilatory response to PDE5i, while the presence of severe mitral or tricuspid regurgitation was related to a more pronounced increase in CO. These findings might help to identify those patients with a possible favorable acute hemodynamic response to PDE5i in particular settings (i.e., LVAD or heart transplant aiming to reduce the risk of acute RHF) and may lead to actions to improve PDE5i responsiveness by increasing plasma potassium to the high range of normality.

Acute hemodynamic effects of PDE5i result from their ability to increase cGMP levels in vascular smooth muscle cells, thereby reducing vascular resistances and pressures (11, 28). Our study indicates that these effects may be modified by plasma potassium level and attenuated by a concentration of this cation in the lower range of normality. Potassium abnormalities are common in HF and are linked with excess morbidity, the risk for arrhythmia, and mortality (29, 30). Interestingly we showed, for the first time, that plasma potassium concentration was inversely associated with pulmonary vascular reactivity after acute PDE5i administration. Whether this is a mere coincidence or plasma potassium level is mechanistically involved in PVR reversibility cannot be sorted out from current data, although the latter is possible. As shown in our study, a lower potassium concentration could be also correlated with the use of high doses of loop diuretics, a typical feature of more advanced HF (i.e., increasing degree of backward RV failure) and, in turn, exhausted pulmonary vasoreactivity. A concentration of potassium in the lower range might increase transmembrane gradient (hyperpolarization) and reduce smooth muscular cell excitability, leading in turn to a blunted pulmonary vasodilatory response after PDE5i (31). Several studies identified voltage-gated potassium channels as key regulators of PA smooth muscular cell excitability (32). Two potassium channels largely represented in lungs, the potassium channel subfamily K member 3 (KCNK3) and the adenosine triphosphate (ATP)-sensitive potassium channels (KATP), are activated by protein kinase G-dependent phosphorylation, suggesting that their modulation might contribute to the vasorelaxant effects of PDE5i (33). This mechanism probably also applies to the systemic circulation, as demonstrated in our study by the attenuated SVR reduction after PDE5i among patients with a concentration of serum potassium in the lower range.

Another moderately good predictor of larger PVR reduction after PDE5i was plasma aldosterone level. Previous human and experimental studies showed that aldosterone concentration is increased in PH and correlated with cardio-pulmonary hemodynamic indexes and pulmonary vascular remodeling (34, 35). In PAH models, aldosterone reduces nitric oxide (NO) availability through increasing pulmonary endothelial oxidant stress (35), which promotes endothelial dysfunction. The administration of PDE5i may reverse this condition by reducing the GMP-mediated NO degradation and improving in turn pulmonary vasoreactivity. Accordingly, higher aldosterone concentrations might directly contribute to the blunted vasodilatory response after PDE5i showed in our study. Altogether, our findings provide new data to support the role of aldosterone signaling in mediating pulmonary vascular dysfunction among patients with advanced HF.

It was proposed that increased PVR may prevent fluid leak in the pulmonary capillary bed and consequent edema formation in patients with PH (36). Accordingly, it was suggested that pulmonary vasodilators may increase the risk of lung congestion in patients with HF (37). Our study challenges these concerns since sildenafil infusion mostly reduced both PVR and PAWP. As previously demonstrated by our group (15), while PVR reduction is a direct effect of PDE5i on pulmonary vasculature, the drop in PAWP is partly due to the diminished pericardial constraint as a consequence of the reduction of RV size. This mechanism is consistent with known contributions of external pericardial force to the LV filling pressure in patients with failing dilated hearts (38). Other mechanisms, such as reduction of venous return, a decrease of LV afterload, and improvement of LV/RV filling pressures with consequent improvement of paradoxical septal motion, could be involved in the observed drop in PAWP after PDE5i. About a fifth of our patients treated with sildenafil showed a rise rather than a reduction in PAWP. This condition was associated with higher BNP and more severe tricuspid regurgitation, possibly identifying a subset of sicker patients, with higher filling pressures and less prone to reduce RV size in response to sildenafil (and consequently with higher pericardial constraint). Lower aldosterone was also found to be a predictor of increased PAWP after sildenafil. This result is in line with responsiveness to PDE5i in terms of PVR and could be related to the same mechanism (i.e., higher PVR response due to lower aldosterone level could unveil an increase in PAWP after sildenafil).

The described profound PDE5i-induced afterload-reducing effects on both pulmonary and systemic circulation could probably also explain the showed moderate association between the severity of mitral regurgitation and the increase in CO. In chronic severe mitral regurgitation, a large portion of stroke volume regurgitates back into the atrium, and the effective forward flow is curtailed and primarily determined by SVR (39). PDE5i administration reduced SVR, resulting in a significant increase in CO and in a consequent mitigation of mitral regurgitation severity (40). The same mechanism applies to the right side for hemodynamic relevant tricuspid regurgitation.

Different magnitude in response to PDE5i among subjects with HF may also come from a heterogeneity in PDE5 expression in the pulmonary vasculature. Indeed, it has been demonstrated that PDE5 isoform expressions were significantly increased in neomuscularized distal vessels and in smooth muscle cells of the medial layer of the diseased pulmonary vasculature (41). Accordingly, our patients with more severe PH seem to show a better hemodynamic response after PDE5 inhibition.

From a clinical perspective, our findings are hypothesis generating and might have relevant implications in the field of advanced HF. The most relevant insight is that PDE5i response seems to be associated with plasma potassium concentration. Indeed, a lower concentration of this electrolyte, even if in the normal range, could be a marker of poor response to PDE5i. In line, clinicians should be aware of a possible blunted pulmonary hemodynamic response to PDE5i when plasma potassium is close to the lower limit of normal. This finding assumes particular relevance in patients with HFrEF with severe PH candidates for LVAD or heart transplant, where the effect of pre-operative treatment with PDE5i could be improved by potassium level modulation (i.e., keeping the plasma potassium in the higher range of normality). Increased aldosterone concentration was also associated with attenuated response to PDE5i, suggesting a potential role of simultaneous use of mineralocorticoid antagonists and PDE5i to enhance PVR reduction in patients with HF and PH (42, 43).

Our analysis has some limitations. We do not have a placebo control group; however, it is unlikely that reported changes after PDE5i could be explained by the period or placebo effects. Plasma potassium and aldosterone levels are interconnected through a negative feedback mechanism; therefore, links between attenuated PDE5i response and low potassium or high aldosterone might be interdependent. Cardiac mechanics was assessed only at a steady state, without manipulation of pre-load. We did not study patients with mild HFrEF or patients with heart failure with mildly reduced ejection fraction (HFmrEF)/heart failure with preserved ejection fraction (HFpEF), and consequently, our findings cannot be extended to all spectra of HF. Due to the small number of patients, we cannot make any inference about the effect of PDE5i on specific subgroups. Individual variability in pharmacodynamics and pharmacokinetics or other unrevealed factors that might affect the response to sildenafil cannot be fully addressed in our study. In our population, virtually all patients showed a potassium level within the normal range (3.5–5.0 mmol/L); consequently, we cannot make any inference about the effect of hypo/hyperkaliemia on pulmonary vasodilatory response to PDE5i. Finally, differences in potassium or salt balance and/or circadian factors that could influence plasma levels of aldosterone and other hormones were not measured in this study.

## Conclusion

We identified, for the first time, a possible role of plasma potassium level in the modulation of pulmonary vascular reactivity after acute administration of PDE5i. Our study is hypothesis generating; therefore, the causality and clinical relevance of these findings should be further investigated.

## Data Availability Statement

The raw data supporting the conclusions of this article will be made available by the authors, without undue reservation.

## Ethics Statement

The studies involving human participants were reviewed and approved by IKEM Institutional Review Board. The patients/participants provided their written informed consent to participate in this study.

## Author Contributions

LM and VM wrote the manuscript and analyzed the data. AR, HA-H, IJ, and ZH conducted the clinical studies, collected the data, and contributed to the manuscript. JK provided critical comments to the manuscript. All authors have participated in the work and have reviewed and agreed with the content of the article.

## Conflict of Interest

The authors declare that the research was conducted in the absence of any commercial or financial relationships that could be construed as a potential conflict of interest.

## Publisher’s Note

All claims expressed in this article are solely those of the authors and do not necessarily represent those of their affiliated organizations, or those of the publisher, the editors and the reviewers. Any product that may be evaluated in this article, or claim that may be made by its manufacturer, is not guaranteed or endorsed by the publisher.

## Supplementary Material

Supplementary Figure 1 Correlation between baseline plasma aldosterone and potassium level.
