# Supplementary material for: Pulmonary Vasculature Responsiveness to Phosphodiesterase-5A Inhibition in Heart Failure With Reduced Ejection Fraction: Possible Role of Plasma Potassium
Source: Front Cardiovasc Med. 2022 May 26;9:883911. doi: 10.3389/fcvm.2022.883911 (PMC9204350; doi:10.3389/fcvm.2022.883911)
Supplement: Supplementary file 1 [file Table_1.docx]

**Supplemental Table 1.** Correlation between baseline clinical variables and PAWP increase after sildenafil.

| **Parameters** | **Odds ratio [95% CI]** | **χ2** | **p-value** |
| --- | --- | --- | --- |
| Age, years | 1.01 [0.94; 1.08] | 0.11 | 0.733 |
| Body mass index, kg/m^2^ | 0.90 [0.74; 1.10] | 0.96 | 0.325 |
| NYHA class, I-IV | 0.68 [0.15; 0.92] | 0.26 | 0.607 |
| HF duration, years | 1.02 [0.90; 1.15] | 0.12 | 0.723 |
| Sodium, mmol/L | 1.15 [0.92; 1.44] | 1.57 | 0.209 |
| Potassium, mmol/L | 5.19 [0.67; 40.25] | 2.89 | 0.088 |
| BNP, ng/L | 3.85 [1.03; 14.38] | 5.45 | 0.019 |
| Aldosterone, pmol/L | 0.17 [0.04; 0.82] | 6.76 | 0.009 |
| Renin, ng/L | 1.02 [0.55; 1.89] | 0.01 | 0.926 |
| PAWP, mmHg | 0.93 [0.85; 1.02] | 1.85 | 0.173 |
| PVR, WU | 1.10 [0.72; 1.67] | 0.20 | 0.653 |
| PA pressure (mean), mmHg | 0.97 [0.90; 1.06] | 0.33 | 0.561 |
| RV-EDP, mmHg | 1.03 [0.87; 1.22] | 0.16 | 0.687 |
| RV maximum pressure, mmHg | 0.96 [0.92; 1.02] | 1.67 | 0.195 |
| LVEF, % | 0.96 [0.82; 1.13] | 0.17 | 0.674 |
| TAPSE, mm | 0.98 [0.82; 1.18] | 0.02 | 0.884 |
| Mitral regurgitation, grade 1-4 | 0.97 [0.50; 1.88] | 0.01 | 0.941 |
| Tricuspid regurgitation, grade 1-4 | 2.76 [1.19; 6.37] | 6.45 | 0.011 |
| Furosemide daily dose, mg/24h | 0.98 [0.97; 1.00] | 3.30 | 0.068 |

**Abbreviations:** BNP – Brain Natriuretic Peptide; HF – Heart Failure; LVEF – Left Ventricular Ejection Fraction; NYHA – New York Heart Association; PA – Pulmonary Artery; PAWP – Pulmonary Artery Wedge Pressure; PVR, Pulmonary Vascular Resistance; RV – Right Ventricle; RV-EDP – Right Ventricular End-Diastolic Pressure; TAPSE – Tricuspid Annular Plane Systolic Excursion; WU – Wood Units
