# Supplementary figures and images for: Pulmonary Vasculature Responsiveness to Phosphodiesterase-5A Inhibition in Heart Failure With Reduced Ejection Fraction: Possible Role of Plasma Potassium
Source: Front Cardiovasc Med. 2022 May 26;9:883911. doi: 10.3389/fcvm.2022.883911 (PMC9204350; doi:10.3389/fcvm.2022.883911)

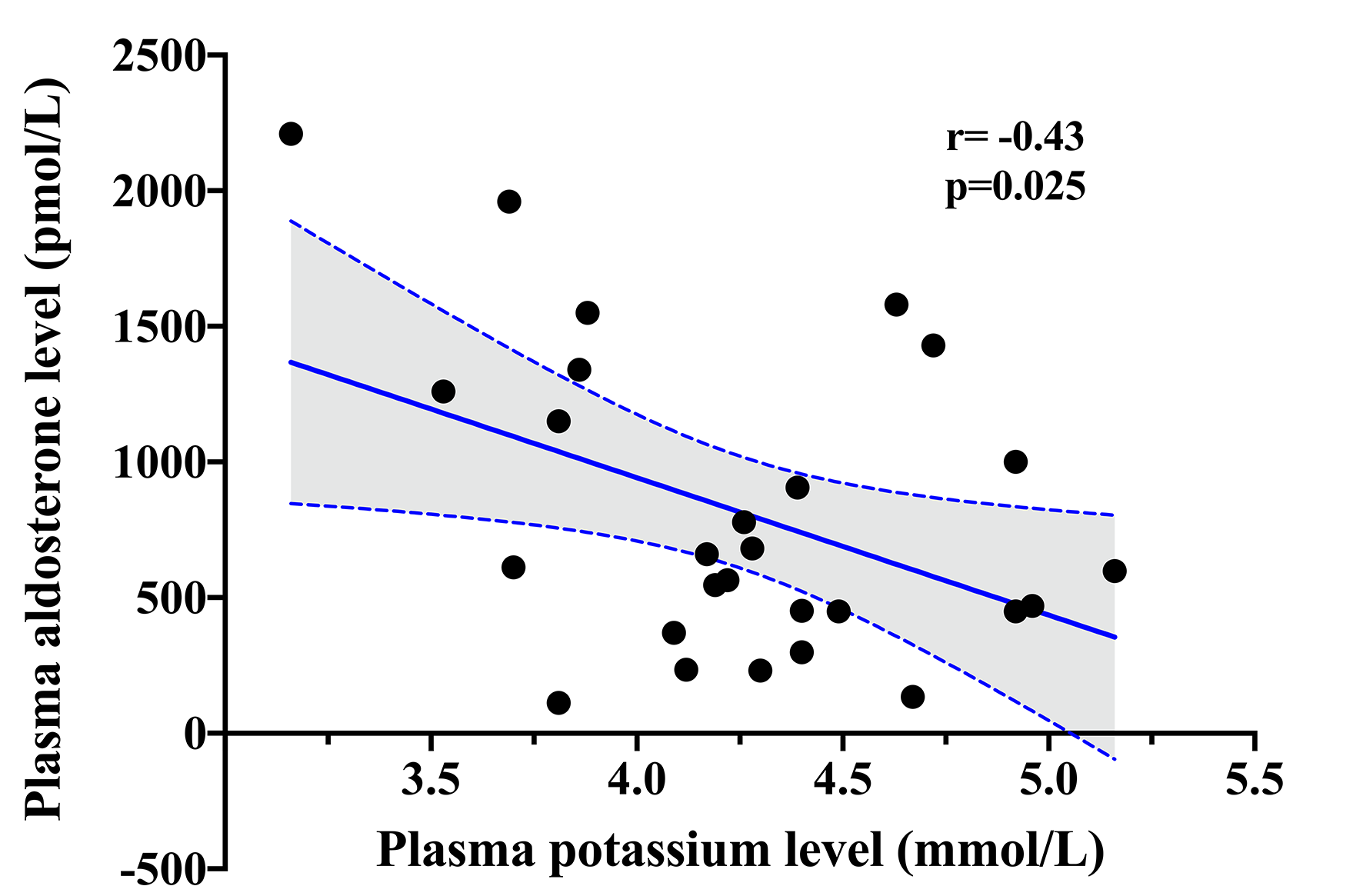

Supplement: Supplementary Figure 1 — Correlation between baseline plasma aldosterone and potassium level. [file Image_1.TIFF]
